# Is *Didymosphenia geminata* an introduced species in New Zealand? Evidence from trends in water chemistry, and chloroplast DNA

**DOI:** 10.1002/ece3.3572

**Published:** 2017-12-12

**Authors:** Cathy Kilroy, Phil Novis

**Affiliations:** ^1^ National Institute of Water and Atmospheric Research Ltd Christchurch New Zealand; ^2^ Landcare Research Lincoln New Zealand

**Keywords:** Chloroplast DNA, *Didymosphenia geminata*, dissolved inorganic nitrogen, dissolved reactive phosphorus, invasive species, New Zealand, nuisance algae, ubiquity hypothesis

## Abstract

Defining the geographic origins of free‐living aquatic microorganisms can be problematic because many such organisms have ubiquitous distributions, and proving absence from a region is practically impossible. Geographic origins become important if microorganisms have invasive characteristics. The freshwater diatom *Didymosphenia geminata* is a potentially ubiquitous microorganism for which the recent global expansion of nuisance proliferations has been attributed to environmental change. The changes may include declines in dissolved reactive phosphorus (DRP) to low levels (e.g., <2 mg/m^3^) and increases in dissolved inorganic nitrogen (DIN) to >10 mg/m^3^ because both these nutrient conditions are associated with nuisance proliferations of *D. geminata*. Proliferations of *D. geminata* have been observed in South Island, New Zealand, since 2004. We aimed to address the ubiquity hypothesis for *D. geminata* in New Zealand using historical river water nutrient data and new molecular analyses. We used 15 years of data at 77 river sites to assess whether trends in DRP or DIN prior to the spread of *D. geminata* were consistent with a transition from a rare, undetected, species to a nuisance species. We used new sequences of chloroplast regions to examine the genetic similarity of *D. geminata* populations from New Zealand and six overseas locations. We found no evidence for declines in DRP concentrations since 1989 that could explain the spread of proliferations since 2004. At some affected sites, lowest DRP occurred before 2004. Trends in DIN also did not indicate enhanced suitability for *D. geminata*. Lack of diversity in the chloroplast intergenic regions of New Zealand populations and populations from western North America is consistent with recent dispersal to New Zealand. Our analyses did not support the proposal that *D. geminata* was historically present in New Zealand rivers. These results provide further evidence countering proposals of general ubiquity in freshwater diatoms and indicate that, as assumed in 2004, *D. geminata* is a recent arrival in New Zealand.

## 1 INTRODUCTION

Species introductions are a recognized component of human‐mediated global change (Ricciardi, 2007; Vitousek, D'Antonio, Loope, Rejmanek, & Westbrooks, 1997). Both deliberately and inadvertently introduced species include those that are considered invasive because of their detrimental impacts on indigenous ecosystems in their new locations (e.g., Leung, Finnoff, Shogren, & Lodge, 2005). The geographic origins of non‐native, invasive, macroorganisms are often known, as are those of plant and animal‐borne pathogenic microorganisms (but see Caudill & Caudill, 2016). Establishing the geographic origins and distributions of free‐living microorganisms (invasive or otherwise) can be more problematic (Van de Vijver, Kelly, Blanco, Jarlman, & Ector, 2008; Vanormelingen, Verleyen, & Vyverman, 2008). Microorganisms frequently have ubiquitous distributions because their small size and often large populations facilitate movement over long distances (Finlay & Clarke, 1999). This “ubiquity hypothesis” is based on the well‐known tenet that, for microorganisms, “Everything is everywhere, but the environment selects” (Baas‐Becking, 1934). However, identification of distinctive freshwater diatom taxa with restricted distributions alongside truly cosmopolitan taxa (Vanormelingen et al., 2008) reinforces a view that everything is *not* everywhere (even if the environment is suitable) (O'Malley, 2008) and that the distributions of microorganisms that have different dispersal and survival capabilities cannot be expected to conform to any consistent patterns (Novis, Beer, & Vallance, 2008). A fundamental problem is that proof of non‐native status for any microorganism requires demonstration of the absence of viable propagules from a region, which is practically impossible using conventional survey methods (Finlay, Monaghan, & Maberly, 2002).

Ability to determine whether a species is native or non‐native becomes important when other components of global change, such as shifts in nutrient pools associated with widespread agriculture and changes in river flows linked to both water diversions and climate change (Foley et al., 2005), create conditions that favor proliferations of particular microorganisms. In this situation, is it possible to identify whether an apparently invasive microorganism new to an area really has been introduced, or is a native taxon that was previously extremely rare? The implications of this question for freshwater management have been highlighted recently by the example of *Didymosphenia geminata* (Lyngbye) Mart. Schmidt (Taylor & Bothwell, 2014).

**Figure 1:**
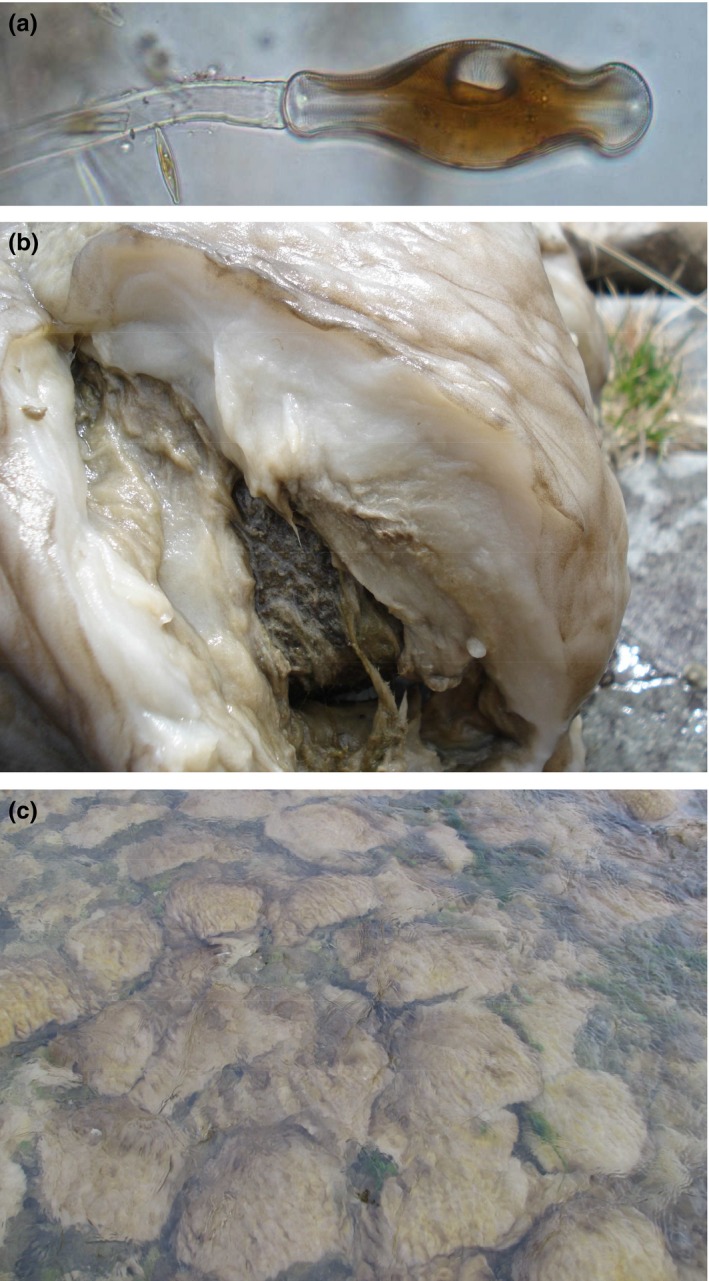
(a) Cell of *Didymosphenia geminata*, with stalk (cell approx. 130 µm long). (b) *D. geminata* mat cut open to show the thick mass of white stalk material with a thin layer of cells at the surface. (c) High cover of a riverbed by *D. geminata* mats

*Didymosphenia geminata* is a large, stalked freshwater diatom that inhabits low‐nutrient streams and rivers and is thought to be native to boreal and mountainous regions of the Northern Hemisphere (Blanco & Ector, 2009). The cells attach to surfaces in rivers with adhesive extracellular polymeric substance (EPS, Wetherbee, Lind, Burke, & Quatrano, 1998) then exude an EPS stalk (Figure 1a). Following cell division, each daughter cell produces a stalk so that colonies eventually comprise bifurcating stalks with a layer of cells at the colony surface (Figure 1b). *D. geminata*'s ability to form high biomass (Figure 1c) in low‐nutrient rivers appears to result from excessive EPS production in nutrient‐limiting conditions (Bothwell & Kilroy, 2011; Bothwell, Taylor, & Kilroy, 2014; Kilroy & Bothwell, 2011, 2012). The proposed mechanism is photosynthetic “overflow” production of carbohydrate when insufficient phosphorus is available to sustain cell division, but light levels favor photosynthetic activity (Staats, Stal, de Winder, & Mur, 2000). In New Zealand, the dissolved reactive phosphorus (DRP) threshold below which proliferations can form appears to be approximately 2 mg/m^3^ (Bothwell et al., 2014; Kilroy & Bothwell, 2012). *D. geminata* is therefore of particular concern because its nuisance proliferations occur in oligotrophic waters, in contrast to other algal blooms, which are normally associated with excessive cell growth under the influence of high nutrients (e.g., Dodds & Smith, 2016; Graham, Graham, & Wilcox, 2009, p. 586). High benthic algal biomass dominated by *D. geminata* may adversely affect the recreational values of rivers (Beville, Kerr, & Hughey, 2012) and alter the structure of higher trophic levels (Jellyman & Harding, 2016).

Over the past three decades, proliferations of *D. geminata* have become more common worldwide including in areas where the species was previously rarely or not reported. Initially, the explanation for the novel occurrence of these blooms was the human‐mediated spread of an invasive species (Bothwell, Lynch, Wright, & Deniseger, 2009). More recently, based on the proposal that very low DRP concentrations are a primary cause of *D. geminata* proliferations (Bothwell et al., 2014), Taylor and Bothwell (2014) hypothesized that the range expansion of *D. geminata* blooms since the 1980s was largely a result of environmental change, specifically, declining DRP in rivers, or oligotrophication (Eimers, Watmough, Paterson, Dillon, & Yao, 2009; Stockner, Rydin, & Hyenstrand, 2000). This view was consistent with observations from Vancouver Island, Canada, from where there are records of *D. geminata* in the 1880s but no reports of proliferations until the 1980s (Bothwell et al., 2009). The first reports of *D. geminata* blooms on Vancouver Island followed a major nitrogen forest fertilization program (Bothwell et al., 2014). Application of N in the catchment would have led to increased terrestrial assimilation of P and, consequently, reduced export of P to waterways. However, no DRP concentration data were presented to support the hypothesis and there has been subsequent debate over its applicability (Bergey & Spaulding, 2015; Keller, Hilderbrand, Shank, & Potapova, 2017; Taylor & Bothwell, 2015). Robust environmental data either supporting or refuting this ubiquity hypothesis for *D. geminata* are still lacking.

*Didymosphenia geminata* proliferations were first reported in New Zealand in October 2004 in the Waiau River catchment (Southland, South Island) (Kilroy, Snelder, Floerl, Vieglais, & Dey, 2008). Since 2004, several lines of circumstantial evidence supported the view that *D. geminata* is a recent introduction to New Zealand. These include lack of reliable prior records of this very distinctive species either from living or fossil diatom collections, and the pattern of spread (Kilroy & Unwin, 2011). Nonindigenous status of *D. geminata* in New Zealand is widely accepted (e.g., Keller et al., 2017). Nevertheless, the Taylor and Bothwell (2014) hypothesis has exposed the possibility that *D. geminata* has been historically present in New Zealand as an extremely rare species.

Here, we present the results of new analyses that address the ubiquity hypothesis for *D. geminata* and the probability of its presence in New Zealand well before the first proliferations were discovered in 2004. Two approaches were taken. First, we analyzed data on DRP and dissolved inorganic nitrogen (DIN), which have been collected from a suite of 77 New Zealand rivers since 1989. The data were used to test the hypothesis that the novel appearance of *D. geminata* proliferations in New Zealand can be linked to declines in DRP to concentrations that favor proliferations. DIN concentrations were also investigated because it has been suggested that low‐level increases in DIN in rivers may also stimulate *D. geminata* proliferations, provided DRP concentrations are sufficiently low (Kilroy & Larned, 2016). Kilroy and Larned (2016) suggested that, in New Zealand, *D. geminata* requires DIN concentrations >10 mg/m^3^ for proliferations to form. Second, we confirmed genetic findings from the nuclear ITS region of *D. geminata* (Kelly, 2009) that populations of *D. geminata* from New Zealand and overseas are genetically very similar, using new sequences of chloroplast intergenic regions. We used the combined environmental and molecular information, along with existing circumstantial evidence (Kilroy & Unwin, 2011), to reassess the probability of presence of *D. geminata* in New Zealand prior to the first discovery of proliferations in 2004.

## 2 METHODS

### 2.1 Trends in nutrient concentrations and relationships with *D. geminata* cover

**Figure 2:**
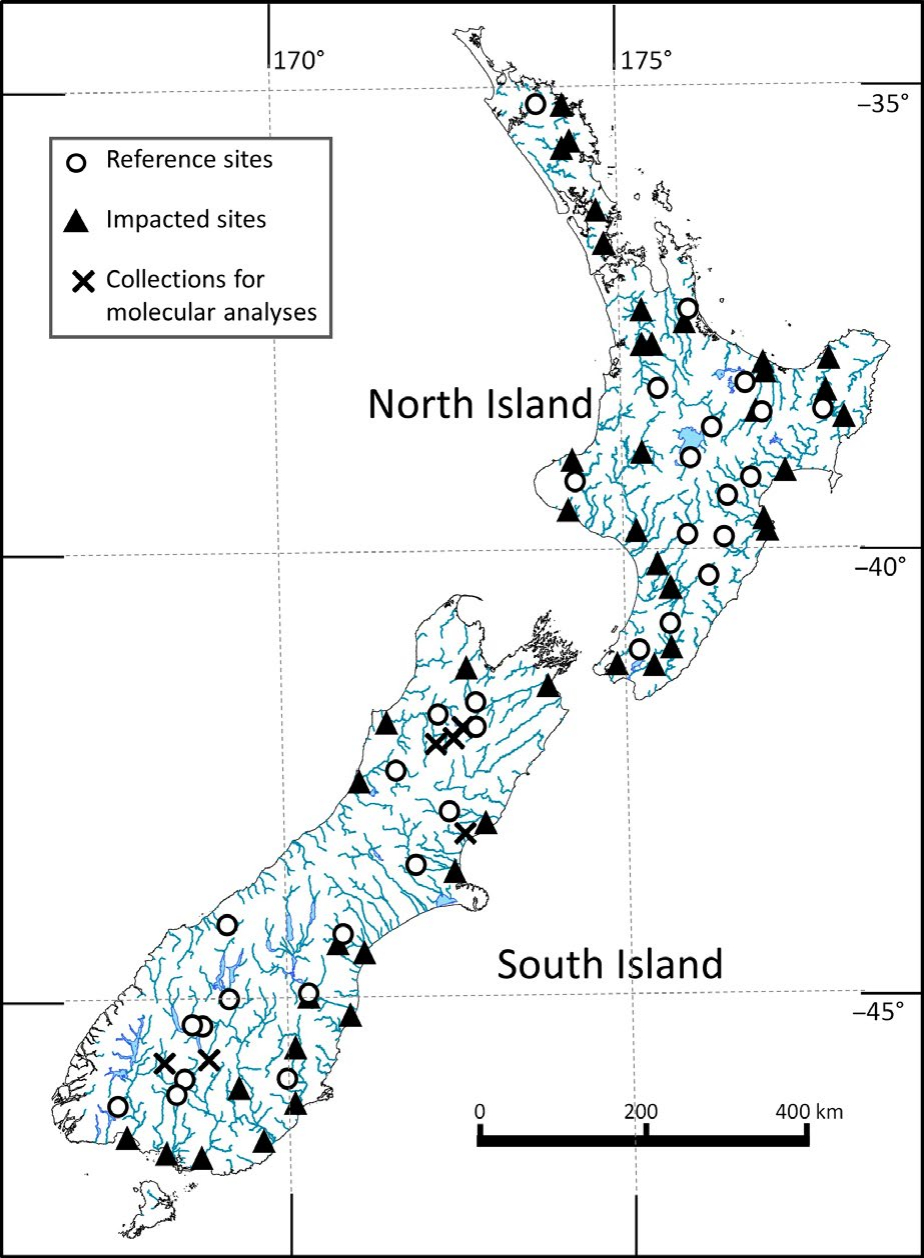
Locations of the 77 NRWQN sites in the North and South Islands of New Zealand showing reference and impacted sites. The locations of sample collections for the molecular analysis of New Zealand material are also shown. Rivers shown are stream order 5 or greater. Refer to Table S1 for coordinates and details for each site

New Zealand's National River Water Quality Network (NRWQN) dataset comprises data on flow, water quality, and estimated periphyton percentage cover collected every month at 77 river sites from throughout the North and South Islands (Figure 2, Table S1). The dataset includes 32 reference sites, with largely undeveloped catchments. The remaining 45 sites (termed impacted hereafter) have varying amounts of urban or agricultural development in their catchments. Data collection commenced in January 1989 (Davies‐Colley et al., 2011) and is ongoing at most sites.

#### 2.1.1 Field and laboratory methods

Periphyton cover (including *D. geminata*) was assessed at each site using visual estimates of percentage cover by periphyton mats and filaments at 10 points along one or two transects in wadeable water depths. Prior to 2012, *D. geminata* presence was reported in field notes, which allowed estimation of mean percentage cover on each survey**.** After January 2012, cover by *D. geminata* was recorded as a separate category. We used the monthly mean cover data from each site and also extracted maximum annual cover (January to December). Sites were assigned to one of four groups according to the *D. geminata* cover eventually recorded the following: (1) sites with persistent cover (visible cover recorded every year since first observed at the site, with >20% cover in at least half of the years); (2) occasional visible but not persistent cover (visible cover recorded in five or fewer years with maximum cover >20% in no more than two of the years); (3) negligible visible cover (cover <1%, or not recorded, but presence confirmed from microscopic examination of samples); (4) not detected.

As part of the NRWQN monitoring, a 1‐L water sample was collected at each site, filtered within 24 h of collection through 0.45‐μm cellulose membrane filters, and analyzed for NO_3_‐N, NH_4_‐N, and DRP using a Lachat QuikChem FIA+ 8000 series analyzer (Lachat Instruments, Milwaukee, WI, USA). Analytical detection limits were approximately 0.5, 1.8, and 0.5 mg/m^3^ for NO_3_‐N, NH_4_‐N, and DRP, respectively. DIN was calculated as the sum of NO_3_‐N and NH_4_‐N.

#### 2.1.2 Data analysis

We first inspected plots of 12‐month moving averages of DRP (geometric means) from 1990 to 2015 alongside monthly *D. geminata* percentage cover. We then assessed the direction of trends in DRP and DIN for the period up to 2006, when *D. geminata* was starting to become widespread in the South Island. We followed the method of McBride, Cole, Westbrooke, and Jowett (2014), using both raw and flow‐adjusted data. Adjustment for the effects of flow is appropriate for nutrient concentrations because, where concentrations increase with flow as nutrients are introduced to rivers in runoff, transient changes in concentrations may mask those resulting from wider catchment changes. Adjusted data were calculated as follows:

adjusted value=raw value−smoothed value + median value.

where the smoothed value is the value predicted by a LOWESS smoother curve fitted to the nutrient vs. flow data (Smith, McBride, Bryers, Wisse, & Mink, 1996). The data series began in 1995 for DIN, because NH_4_‐N data were unreliable for the first five years of the monitoring program (Davies‐Colley et al., 2011). For DIN, we focussed on reference sites, which included all sites with mean DIN low enough to preclude development of *D. geminata* mats (i.e., <10 mg/m^3^, Kilroy & Larned, 2016).

Trends were calculated using the Seasonal Sen Slope estimator, which calculates the median of slopes between all pairs of data points within each month. The importance of the trend was determined by calculating a symmetric 100 (1–2α) % confidence interval on the Sen Slope. Evidence for *any* trend up or down was inferred if the confidence interval did not contain zero. If the interval contained zero, the interpretation was that there were insufficient data to determine the trend direction. All trend analyses were performed using the freeware TimeTrends v. 5.0, http://www.jowettconsulting.co.nz/home/software.

The dataset was divided into periods prior to widespread occurrence of *D. geminata* (up to and including 2005) and following its widespread occurrence in multiple South Island rivers (2006 to 2015). The two periods are referred to subsequently as the pre‐ *D. geminata* and post‐ *D. geminata* periods. We compared DRP distributions between the two periods and average annual concentrations within groups of sites based on *D. geminata* abundance. Statistical significance of differences in distributions was determined using two‐sample Kolmogorov–Smirnov tests, and between means using two‐sample *t* tests or ANOVA. Data were log‐transformed to ensure homogeneous variance. At sites with persistent *D. geminata* cover in the post‐ *D. geminata* period, we used linear regression to assess relationships between maximum annual percentage cover and mean annual DRP over the years since establishment of *D. geminata*.

### 2.2 Molecular analyses

DNA was extracted from the samples shown in Table S2 (see Figure 2 for locations) as described by Novis, Schallenberg, and Smissen (2015).

The published chloroplast genome of *D. geminata* (Genbank accession KC509523) was used to design primers spanning intergenic regions in both the large and small single‐copy regions of the genome. Sequences of amplicons from three primer sets are reported here (Table 1), corresponding to the regions between atpF–atpH, rbcS–rbcL, and secA–rbcR. These primers were designed such that the 3′ end of one of each pair overlapped the spacer region, in order to increase specificity and avoid cloning from environmental samples. PCR was run according to the following conditions: 94°C for 4 min, followed by 35 cycles of 94°C for 30 s, 57–60°C (depending on the sample) for 30 s, and 72°C for 45 s. Products were visualized on agarose gels using ethidium bromide staining. Sequencing was carried out by Landcare Research, Auckland, New Zealand, using BDT 3.1 (Applied Biosystems, Foster City, CA, USA). Amplifications were successful for all three regions in all samples except for the sample from Norway, in which the rbcS–rbcL failed to amplify. This region was included in the dataset as missing data. All electropherograms were of high quality apart from the rbcS–rbcL sequence of the Iranian sample. This sequence was estimated from the trace with assistance from other *Didymosphenia* sequences for the same region, but may contain some errors. As *Didymosphenia* from this site was shown to be the most distant from the New Zealand samples according to the other regions (sequence differences and intron presence), this would have minimal effect on the analysis.

**Table 1:** Sequences and binding sites of primers used in this study

Primer name	Sequence (5′‐3′)	Binding site^a^
atpF–atpH F	GCTGCTGGTTTAGCTATTGG	54,754
atpF–atpH R	AAATTTTCCATGATTTCGAG	55,688
rbcR–secA F	GTTAATGCAAATGACTCAGC	108,632
rbcR–secA R	CTTAGAAAGTTGAAAGATCG	109,526
rbcS–rbcL F	GTCTCACTATTCAATACTCC	49,777
rbcS–rbcL R	TGTATGGAAGGTATTAACCG	50,584

a According to the published chloroplast genome sequence of *D. geminata*, Genbank accession KC509523.

Multiple sequence alignment was carried out using MEGA version 6 (Tamura, Stecher, Peterson, Filipski, & Kumar, 2013) and checked by eye. The resulting dataset contained 12 sequences and 2230 sites, 14 of which were variable (2 parsimony‐informative), not counting a 53 base pair insertion unique to the Iranian sample. Polymorphic sites were triple‐checked on electropherograms due to the low diversity present in the sequences. We elected to present the results as numbers of differences and p‐distances, also as a result of this low diversity.

## 3 RESULTS

### 3.1 Trends in nutrient concentrations and relationships with *D. geminata* cover

**Figure 3:**
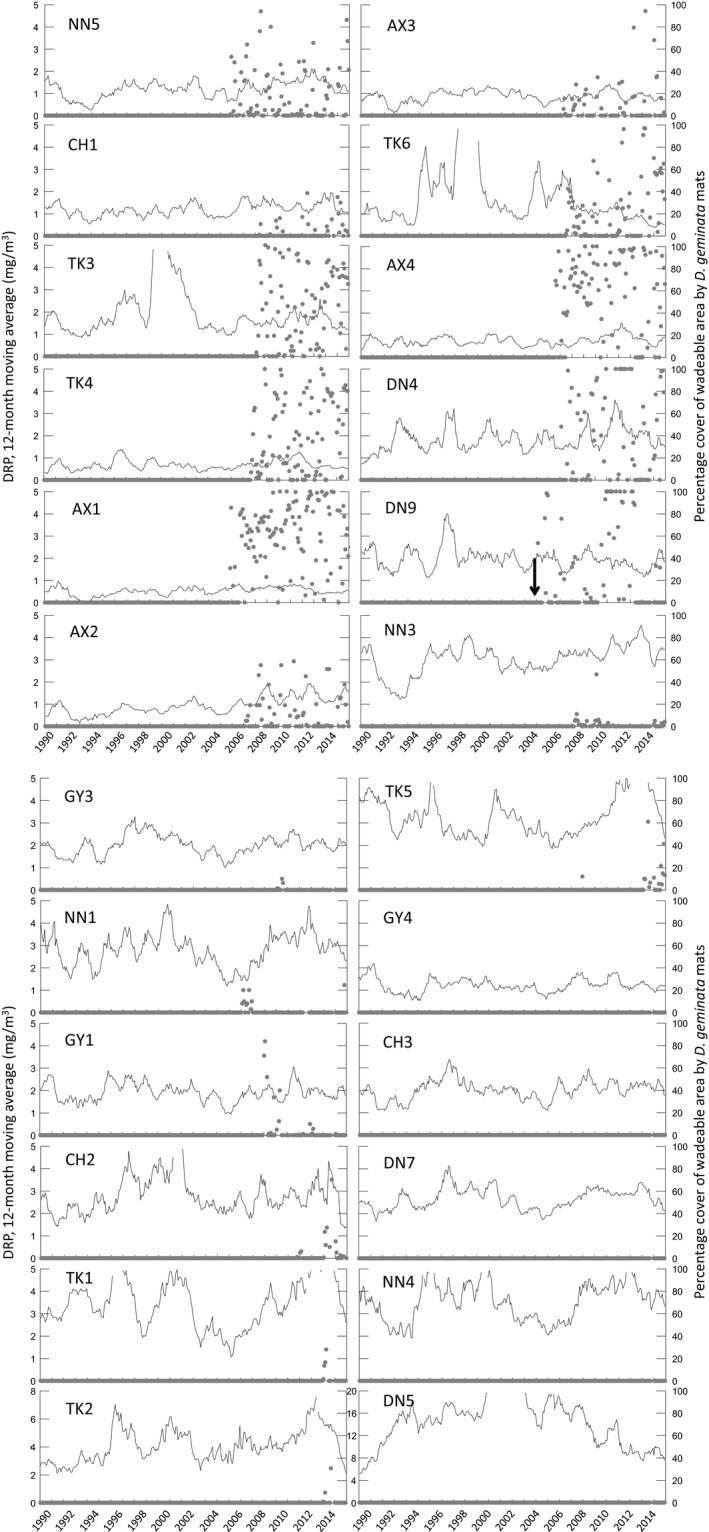
Twelve‐month moving averages of DRP plotted against time at the 24 NRWQN South Island sites at which *Didymosphenia geminata* has been detected in monthly surveys, with overplots of monthly percentage cover by *D. geminata* (gray dots). The arrow on DN9 (first panel, lower right) indicates the time of the first observations of *D. geminata* proliferations in New Zealand, in the Lower Waiau River. Sites are in the same order as in Table 2. Note different scales for DRP at TK2 and DN5 (bottom plots on second panel). At the last five sites, *D. geminata* cover was negligible and is not visible on the plots

Visible *D. geminata* cover was recorded at 18 of the 33 river monitoring sites in the South Island between 2004 and 2008, with persistent cover at 11 sites and occasional cover at seven sites. Rare occurrences of negligible cover (<1%) were detected at a further five sites. (Figure 3, Table 2). No *D. geminata* was detected at 10 sites between 2004 and 2008, but appeared at one of them (TK2) in 2014. No *D. geminata* was observed at any of the 44 sites in the North Island (Table S1).

**Table 2:** Results of analyses of trends in DRP between 1989 and 2006 at 33 river sites in South Island, New Zealand, with sites divided into groups based on abundance and frequency of occurrence of *Didymosphenia geminata* cover between 2005 and 2008 (the main period of spread in New Zealand)

Site code	Site name	State	Mean DRP (mg/m^3^)		Trend analysis, raw data				Trend analysis, flow‐adjusted data				
			1989	2006	Sen slope	5% CI	95% CI	Result	% expl.	Sen slope	5% CI	95% CI	Result
Sites with persistent visible *D. geminata* cover (>20%)													
NN5	Buller at Longford	Reference	1.6	0.8	0.00	−0.01	0.02	ND	30.4	0.01	−0.02	0.02	ND
CH1	Hurunui at Mandamus	Reference	1.2	1.8	0.00	−0.01	0.01	ND	22.8	0.00	−0.01	0.02	ND
TK3	Opuha at Skipton Br	Reference	1.4	1.9	0.00	0.00	0.03	ND	−0.6	0.02	−0.01	0.04	ND
TK4	Waitaki at Kurow	Reference	0.3	0.7	0.00	−0.01	0.00	ND	8.8	0.00	−0.01	0.01	ND
AX1	Clutha at Luggate Br	Reference	0.5	0.7	0.01	0.00	0.02	ND	1.3	0.02	0.01	0.03	Up
AX2	Kawarau at Chards Rd	Reference	0.4	1.1	0.02	0.00	0.03	ND	1.6	0.02	0.01	0.03	Up
AX3	Shotover at Bowens Peak	Reference	0.7	0.8	0.00	−0.01	0.01	ND	33.9	0.01	0.00	0.02	Up
TK6	Waitaki at SH1 Bridge	Impacted	0.9	2.1	0.02	0.00	0.05	ND	−4.8	0.03	0.01	0.06	Up
AX4	Clutha at Millers Flat	Impacted	0.3	0.6	0.00	−0.01	0.01	ND	20.1	0.00	−0.02	0.01	ND
DN4	Clutha at Balclutha	Impacted	0.6	1.4	0.01	0.00	0.03	ND	2.6	0.02	0.00	0.04	ND
DN9	Waiau at Tuatapere	Impacted	2.3	2.0	−0.01	−0.03	0.00	ND	11.6	−0.02	−0.04	0.00	ND
Sites with occasional visible but not persistent *D. geminata* cover (>1 < 20%)													
NN3	Wairau at Dip Flat	Reference	3.0	3.4	0.02	0.00	0.04	ND	28.6	0.02	0.00	0.04	ND
GY3	Grey at Waipuna	Reference	2.1	1.8	0.00	−0.02	0.00	ND	40.8	0.00	−0.02	0.02	ND
NN1	Motueka at Woodstock	Impacted	3.8	1.6	−0.06	−0.11	−0.02	Down	43.8	−0.03	−0.06	0.00	ND
GY1	Buller at Te Kuha	Impacted	1.8	1.6	−0.02	−0.05	0.00	ND	33.7	−0.03	−0.05	−0.02	Down
CH2	Hurunui at SH1 Bridge	Impacted	2.4	3.0	0.04	0.00	0.07	ND	18.7	0.06	0.03	0.09	Up
TK1	Opihi at Waipopo	Impacted	2.8	2.0	−0.07	−0.11	−0.03	Down	38.8	−0.06	−0.09	−0.02	Down
TK2^a^	Opihi at Rockwood	Impacted	2.5	5.5	0.04	0.00	0.08	ND	19.3	0.07	0.03	0.12	Up
TK5	Hakataramea u/s MH Br	Impacted	4.0	2.1	−0.06	−0.10	−0.04	Down	32.1	−0.07	−0.10	−0.04	Down
Sites with negligible *D. geminata* cover (<1%)													
GY4	Haast at Roaring Billy	Reference	1.6	1.1	−0.01	−0.03	0.00	ND	32.5	−0.01	−0.02	0.01	ND
CH3	Waimakariri at Gorge	Reference	1.8	2.6	0.00	−0.01	0.03	ND	33.4	0.03	0.01	0.04	ND
DN7	Oreti at Lumsden	Reference	2.5	2.4	0.00	−0.02	0.01	ND	33.2	0.01	−0.02	0.02	ND
NN4	Wairau at Tuamarina	Impacted	3.7	2.8	−0.03	−0.07	0.00	ND	53.6	−0.03	−0.05	0.01	ND
DN5	Mataura at Seaward Downs	Impacted	5.6	19.0	0.63	0.49	0.76	Up	0.6	0.51	0.79	14.60	Up
Sites where *D. geminata* was not detected													
NN2	Motueka at Gorge	Reference	2.5	2.3	0.00	0	0.02	ND	6.1	0.01	−0.01	0.03	ND
DN2	Sutton at SH87	Reference	4.3	4.5	0.00	−0.05	0.03	ND	3.1	−0.01	−0.05	0.03	ND
DN6	Mataura at Parawa	Reference	3.9	8.3	0.02	−0.01	0.07	ND	6.8	0.03	0	0.06	ND
DN10	Monowai at below Gates	Reference	0.7	0.6	0.00	−0.02	0	ND	2.6	−0.01	−0.02	0	ND
GY2	Grey at Dobson	Impacted	1.8	2.3	0.00	−0.01	0.02	ND	56.2	0.01	−0.01	0.03	ND
CH4	Waimakariri u/s Old Hw Br	Impacted	1.9	2.9	0.00	−0.03	0.02	ND	33.7	0	−0.02	0.02	ND
TK2	Opihi at Rockwood	Impacted	2.5	5.5	0.04	0	0.08	ND	19.3	0.07	0.03	0.12	Up
DN1	Taieri at Tiroiti	Impacted	8.5	15.6	0.40	0.23	0.53	Up	13.9	0.37	0.24	0.51	Up
DN3	Taieri at Outram	Impacted	5.3	9.4	0.04	0	0.13	ND	10.3	0.1	0.02	0.19	Up
DN8	Oreti at Riverton Hwy Br	Impacted	5.3	5.4	0.09	0.03	0.15	Up	27.4	0.14	0.09	0.18	Up

Under Result, ND = trend not detectable from the data. CI = confidence interval; % expl. = the percentage of variance in DRP explained by river flow on the day of sampling.

a At TK2, visible *D. geminata* was first detected in 2014.

Using raw data, no trend in DRP was detectable between 1989 and 2006 at all 11 South Island sites in which persistent *D. geminata* cover ultimately developed (Table 2). Declines in DRP concentrations were detected at three of the eight sites with occasional visible *D. geminata* cover. One site with negligible cover and two sites at which *D. geminata* was not detected showed an increasing trend. Using flow‐adjusted data revealed an upward trend at four sites with persistent *D. geminata* (AX1, AX2, AX3, and TK6) and two with occasional cover (CH2 and TK2), and a downward trend at three sites with occasional cover (GY1, TK1, and TK5) (Table 2).

Across the whole dataset of 77 sites, using flow‐adjusted data, there was evidence for a decline in DRP concentrations at nine sites (12%). Approximately half of all sites showed trends whose direction was not detectable from the data, and 38% an upward trend. The upward trends occurred more frequently at sites classed as impacted rather than at reference sites, particularly in the North Island (Table 3).

**Table 3:** Summary of trends in dissolved reactive phosphorus at 77 river sites in New Zealand, 1989 to 2006. ND = trend not detectable from the data

		Raw data: sites with trend:			Flow‐adjusted data: sites with trend:		
		Down	ND	Up	Down	ND	Up
North Island (*n *= 44)	Reference	3	7	6	4	7	5
	Impacted	3	16	9	2	12	14
South Island (*n *= 33)	Reference	0	16	0	0	13	3
	Impacted	3	11	3	3	7	7
Percentages of all sites							
All sites	%	12	65	23	12	51	38

**Figure 4:**
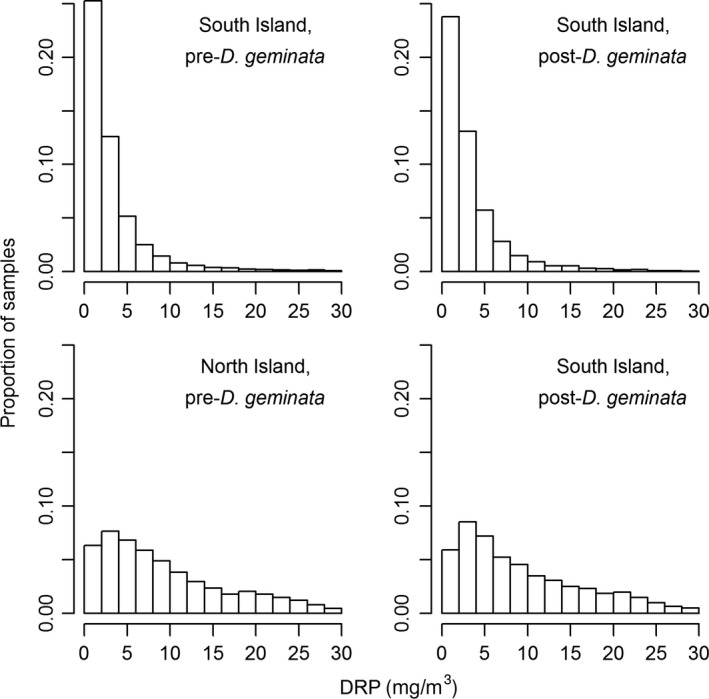
Distributions of DRP observations in the South and North Islands, in the pre‐ and post‐*Didymosphenia geminata* periods. For clarity, DRP concentrations were restricted to ≤30 mg/m^3^. Over 9% and <1% of samples in the South and North Islands, respectively, exceeded 30 mg/m^3^

**Figure 5:**
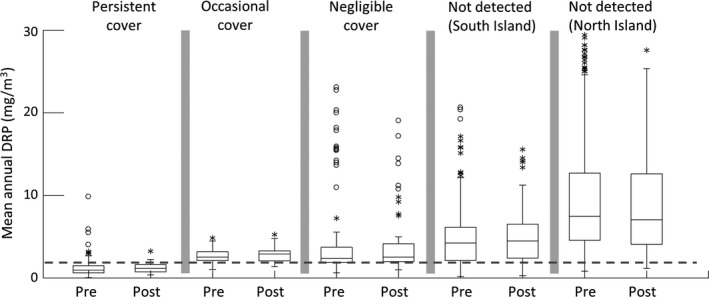
Box plots showing mean annual DRP data at sites grouped according to *Didymosphenia geminata* abundance in pre‐ and post‐ *D. geminata* periods. North Island sites (no *D. geminata* detected at any site) are included as a separate group. Within each group, there was no difference between periods (2‐sample t tests on log‐transformed data, *p *>* *.95 for all comparisons)

The distributions of all DRP data from North and South Island sites in the pre‐ and post‐ *D. geminata* periods appeared similar in the two periods, although K‐S tests showed significant differences (*p* < .001); distributions differed markedly between the North and South Islands (Figure 4). Mean average annual DRP in the pre‐ and post‐ *D. geminata* periods was not significantly different (North Island, 13.0 vs. 11.9 mg/m^3^, respectively; South Island, 3.3 vs. 3.2 mg/m^3^, respectively, two‐sample *t* tests, *p *>* *.25). Average annual DRP also did not differ between pre‐ and post‐ *D. geminata* periods within groups of sites based on *D. geminata* cover (Figure 5). DRP differed between the groups within each period, except between sites with occasional and negligible cover (*p *>* *.1, ANOVA, Tukey's post hoc HSD tests).

Mean annual DRP and maximum annual percentage cover by *D. geminata* were negatively correlated at two sites: mean annual DRP explained 56% (*p *<* *.02, *n *=* *9) and 43% (*p *<* *.05, *n *=* *9) of the variance in annual maximum percentage cover, across ranges of DRP of 0.9–1.9 and 0.8–2.3 mg/m^3^, at NN5 and TK6, respectively.

Between 1995 and 2006, mean DIN concentration ranged from 12 to 1070 mg/m^3^ at the 24 South Island sites where *D. geminata* was eventually recorded, and 12 to 243 mg/m^3^ at sites which had persistent high cover; concentrations were generally higher at the sites classed as impacted (Table S1). Of the 12 reference sites at which *D. geminata* was recorded, there was an upward trend in flow‐adjusted DIN at two sites, both of which have subsequently had negligible cover of *D. geminata*. The trend was down at seven sites, and no trend was detectable at three sites (Table 3). Flow‐adjusted DIN declined at three of the reference sites (NN2, DN2, and DN10) at which *D. geminata* was not detected, and there was no evidence for a trend at a fourth site (DN6, Table 4).

**Table 4:** Trends in dissolved inorganic nitrogen at 16 South Island river sites classed as reference sites (minimal catchment development), with sites divided into groups based on abundance and frequency of occurrence of *Didymosphenia geminata* cover

Code	Site name	Mean DIN (mg/m^3^)		Trend analysis, flow‐adjusted data				Result
		1995	2006	% expl.	Sen slope	5% CI	95% CI	
Sites with persistent *D. geminata* cover (>20%)								
NN5	Buller at Longford	34	19	11.7	−0.38	−0.82	−0.02	Down
CH1	Hurunui at Mandamus	13	11	13.2	0.09	−0.15	0.35	ND^a^
TK3	Opuha at Skipton Br	207	224	6.7	−4.52	−11.14	−0.29	Down^b^
TK4	Waitaki at Kurow	13	5	4.4	−1.23	−1.60	−0.81	Down
AX1	Clutha at Luggate Br	41	32	3.7	−0.69	−1.10	−0.36	Down
AX2	Kawarau at Chards Rd	32	31	14.4	0.18	−0.06	0.30	ND
AX3	Shotover at Bowens Peak	24	17	7.8	−0.37	−0.74	−0.04	Down
Sites with occasional visible but not persistent *D. geminata* cover (>1 < 20%)								
NN3	Wairau at Dip Flat	22	16	24.0	−0.38	−0.63	−0.08	Down
GY3	Grey at Waipuna	23	39	3.3	0.93	0.35	1.78	Up
Sites with negligible *D. geminata* cover (<1%)								
GY4	Haast at Roaring Billy	32	35	−1.5	0.09	−0.32	0.42	ND
CH3	Waimakariri at Gorge	77	62	17.6	−1.07	−1.74	−0.44	Down
DN7	Oreti at Lumsden	295	450	17.5	15.4	8.87	22.4	Up
Sites where *D. geminata* not detected								
NN2	Motueka at Gorge	19	18	0.0	−0.37	−0.74	−0.07	Down
DN2	Sutton at SH	50	10	22.7	−1.45	−2.45	−0.9	Down
DN6	Mataura at Parawa	206	225	36.6	1.11	−0.94	2.88	ND
DN10	Monowai at Below Gates	13	10	26.2	−0.25	−0.42	−0.04	Down

% expl. = the percentage of variance in DRP explained by river flow on the day of sampling. CI = confidence interval. Results are shown for flow‐adjusted data only because flow adjustment revealed most of the trends in DRP. ND = trend not detectable from the data.

a Downward trend using raw data.

b Trend not detectable using raw data.

### 3.2 Molecular analyses

Genetic variation over the chloroplast regions sequenced was very small. All collections of New Zealand material were identical (Table 5). These were also identical to material collected from Vancouver Island (Canada) and Colorado (USA). Small differences (1–3 bp) were found between material from these sites and that collected from River Cocquet (United Kingdom), Boulder Creek (Missouri), and the Nidelva River (Norway). Material from Iran was more divergent from other collections (6–10 bp). In addition, the Iranian material contained a 53 bp insertion in the atpF–atpH intergenic region; this insertion was also largely shared by the species *Gomphoneis minuta* var. *cassieae* (not shown) and was mostly alignable between the two, indicating that its inclusion in the genome represents the ancestral state in this group.

**Table 5:** Matrix of base differences (p‐distances in brackets for differences >0) between *Didymosphenia* collections

Country	Site	Distance matrix										
New Zealand	Gowan River, Nelson											
	Buller River/Howard River, Nelson	0										
	Buller below Lake Rotoiti, Nelson	0	0									
	Hurunui River, Canterbury	0	0	0								
	Wye River, Otago	0	0	0	0							
	Mararoa River at Kiwi Burn, Southland	0	0	0	0	0						
Canada	Little Qualicum River, Vancouver Island	0	0	0	0	0	0					
USA	Boulder Creek, Colorado	0	0	0	0	0	0	0				
	Boulder Creek, Montana	3 (0.001)	3 (0.001)	3 (0.001)	3 (0.001)	3 (0.001)	3 (0.001)	3 (0.001)	3 (0.001)			
United Kingdom	River Coquet	1 (0.000)	1 (0.000)	1 (0.000)	1 (0.000)	1 (0.000)	1 (0.000)	1 (0.000)	1 (0.000)	4 (0.002)		
Norway	Nidelva River, Trondheim	3 (0.002)	3 (0.002)	3 (0.002)	3 (0.002)	3 (0.002)	3 (0.002)	3 (0.002)	3 (0.002)	3 (0.002)	3 (0.002)	
Iran	Tar River	9 (0.004)	9 (0.004)	9 (0.004)	9 (0.004)	9 (0.004)	9 (0.004)	9 (0.004)	9 (0.004)	8 (0.004)	10 (0.005)	6 (0.004)

## 4 DISCUSSION

Although vigorous debate has taken place in the scientific (Bergey & Spaulding, 2015; Taylor & Bothwell, 2014, 2015) and popular literature (Science Media Centre 2014) concerning the role of declining phosphorous in the appearance of problematic blooms of *D. geminata*, empirical data to test the hypothesis of Taylor and Bothwell (2014; that reduced P favors *D. geminata* blooms) have been lacking until now. Bergey and Spaulding (2015) noted that “investigations of patterns of nutrient concentrations relative to blooms are needed”. Here, we have used a long‐term, high‐quality, river monitoring dataset from New Zealand to specifically address this knowledge gap.

Before considering water chemistry, several additional lines of evidence are pertinent to the appearance of *D. geminata* in New Zealand. Taylor and Bothwell (2014) suggested that small populations of *D. geminata* persisting under conditions unfavorable to blooming could have been missed during historical routine monitoring; this is possible but contentious, due to the species’ large, distinctive cells (Bergey & Spaulding, 2015; Kilroy & Unwin, 2011). Furthermore, the biology of *D. geminata*, which has now been subject to considerable investigation, would seem to preclude its long‐distance dispersal to an isolated archipelago without assistance from humans. It is destroyed by exposure to salt, freezing, drying, and the low pH characteristic of a bird gut (Kilroy, Lagerstedt, & Robinson, 2007), thereby restricting its dispersal by wind, ocean currents, or birds. If this is correct, a strict ubiquity hypothesis for *D. geminata* requires a vicariant origin for the species (co‐occurrence in an ancestral landmass that has since split up). However, as the estimated separation of New Zealand from other land masses is >80 Mya (Laird & Bradshaw, 2004), and diverse raphid pennate diatoms (the group including *Didymosphenia*) do not appear in the fossil record until the late Eocene (approx. 50 Mya; Sims, Mann, & Medlin, 2006), a vicariant origin of New Zealand *D. geminata* is not possible.

Molecular genetics of *D. geminata* indicate a much more recent dispersal to New Zealand. DNA sequences accumulate change over time, at a rate depending on the functional constraints (effect on fitness) of the genomic region under consideration, a feature known as a molecular clock (Kumar, 2005). Although molecular clocks are influenced by idiosyncracies of individual lineages, such as generation times, population sizes, and intensity of selection (Ayala, 1999), and divergence estimates are associated with errors (sometimes considerable; Graur & Martin, 2004), isolated populations of *D. geminata* persisting in New Zealand for substantial time periods would accumulate mutations in the intergenic chloroplast regions sequenced by us. In fact, there is almost no diversity in these regions (Table 5), and little more in the ITS region (Kelly, 2009) further indicating recent dispersal. The chloroplast sequence data (being identical within New Zealand and to sequences from Vancouver Island and Colorado) suggest a dispersal event from North America, where a greater genetic diversity within the species occurs, although the limited geographic sampling of this dataset must also be acknowledged. Similar data have been recently used to assert that *D. geminata* is a recent invader to parts of eastern North America (Keller et al., 2017); however, the USA situation differs in having prior records further west (e.g., Letham et al., 2016), with no oceanic dispersal barriers to the eastern seaboard. Our limited sampling shows some diversity within North American specimens (Table 5), as also found by others (Keller et al., 2017). Neither earlier records nor genetic diversity were features of the New Zealand data.

Our dataset, with low sequence diversity, satisfies the common assumptions of chloroplast sequence evolution made in phylogenetics (inheritance as a single nonrecombining linkage, i.e., without intergenomic recombination; Christie & Beekman, 2017; Sullivan, Schiffthaler, Thompson, Street, & Wang, 2017). However, it should be noted that the contributions of different processes shaping the evolution of chloroplast DNA sequences in *D. geminata* are currently uncertain. In particular, it is not clear whether *D. geminata* undergoes a sexual cycle. Size restoration, usually assumed to occur following sexual reproduction, has been clearly demonstrated in *D. geminata* (Bishop & Spaulding, 2017), but the possibility of an asexual mechanism to achieve this has not been ruled out, as neither these authors nor others have observed any other sign of sexual reproduction despite examining many thousands of cells. The mode of inheritance of plastids appears to affect the architecture of their genomes (Crosby & Smith, 2012), and inter‐ and intraspecific recombination has been detected in the plastid genome of *Pseudo‐nitzschia*, another raphid diatom that features biparentally inherited plastids (as might have been expected for *D. geminata*). The enigmatic life cycle of *D. geminata* thus also represents an intriguing unsolved mystery regarding its chloroplast genetics.

Taylor and Bothwell (2014) posited more than ubiquity of *D. geminata*; they also proposed a mechanism to explain its apparent absence in many sites prior to its recent proliferation. Under their model, this species has been globally present for some time, but has only been detected recently in many areas due to lowered phosphorous concentrations in freshwaters.

However, we found no correlation between the timing of the first observations of *D. geminata* proliferations in New Zealand in 2004 and the history of dissolved phosphorous in rivers prior to 2004. At sites where *D. geminata* cover became persistent, no trends in DRP were detected over the 15 years prior to the period during which *D. geminata* started to spread. At the site where *D. geminata* proliferations were first detected (DN9, Waiau River, Kilroy & Unwin, 2011), the complete time series shows that mean DRP had been similar to that in 2004, in 1991–92, and in 1995, yet no *D. geminata* cells or visible cover was detected in the river until 2004 (Kilroy, Larned, & Biggs, 2009). At several other sites, the lowest DRP concentrations recorded occurred prior to detected incursions (e.g., sites NN5, CH1, TK4, TK6, AX1, and AX4). If one assumes the Taylor and Bothwell hypothesis and unconstrained dispersal of *D. geminata* across sites, any one of these sites is enough to refute the hypothesis for New Zealand. Furthermore, across all 77 sites, there was no evidence for a region‐wide decline in DRP concentrations over the period 1989 to 2006, either at reference or impacted sites, or in the North or South Island.

In practice, we expect stochastic effects to operate, even on the distribution and dispersal of microalgae (e.g., Novis et al., 2008), leading to nonuniform dispersal; however, we regard the strong evidence from multiple sites that prolific *D. geminata* growth did not occur at historically lowest DRP concentrations as confirmation of very recent first dispersal of the species to New Zealand.

While lack of consistent declines in DRP up to the time of the first discovery of visible *D. geminata* in a New Zealand support the contention that the species is a recent arrival, the combined DRP and percentage cover data do lend support to the Taylor and Bothwell (2014) hypothesis regarding the association between *D. geminata* proliferations and temporal changes in DRP concentrations. First, up to the end of 2015, *D. geminata* had not been observed at any of the North Island NRWQN sites. Mean DRP concentrations at these sites were higher than those in the South Island (see Table S1), with only 11% of observations at or below the suggested 2 mg/m^3^ threshold for proliferations compared to 49% in the South Island (NRWQN data). Second, annual maximum percentage cover was negatively correlated with DRP at two South Island sites (NN5 and TK6). In addition, visible *D. geminata* at site NN1 was recorded only during dips in DRP concentration below the levels previously recorded, first in 2007 and then in 2015. Lack of relationships between DRP and percentage cover at other sites can be attributed to consistently low mean DRP (<1.2 mg/m^3^) in some cases (e.g., TK4, AX1, AX3, and AX4). Relationships are not expected to always be clear because attachment of *D. geminata* cells to substrata does not appear to be inhibited by elevated DRP, at least in the short term (Kilroy & Bothwell, 2014), and changes in cover under prolonged elevated DRP and stable flows may take months (Kilroy & Larned, 2016).

Nevertheless, correlations we detected between DRP and *D. geminata* percentage cover over time indicate that increased stalk production by *D. geminata* in response to declining DRP concentrations may occur within a year of the start of the decline. In relation to the question of whether *D. geminata* was present in New Zealand rivers prior to 2004, responses to DRP over such time scales suggest that the long periods of DRP <2 mg/m^3^ prior to 2004 at many sites would have been adequate for *D. geminata* proliferations to develop, had the species been present in those rivers.

Detection of correlations between *D. geminata* abundance (or abundance of any alga) and nutrient concentrations at individual river sites over time is difficult because other factors affect temporal changes in both algal standing crop (Biggs, 1996) and nutrient concentrations (Alexander et al., 2009). Therefore, relationships can be uncovered only through the use of long time series (e.g., Suplee, Watson, Dodds, & Shirley, 2012). The present analysis, using nine years of data on *D. geminata* percentage cover, was made possible only by the fortuitous existence of a national, long‐term, river water quality monitoring program that has used consistent and high‐quality methods from the outset (Davies‐Colley et al., 2011).

In addition to DRP, DIN concentrations may influence the establishment and development of *D. geminata* mats. The response by *D. geminata* to DIN appears to be nonlinear. *D. geminata* cover and standing crop have been reported to be negatively correlated with nitrate‐N concentration, although no range of N was stated (Richardson et al., 2014). In New Zealand, proliferations have not been observed in waters with mean DIN >235 mg/m^3^ (Table S1), although low cover or absence at such concentrations may reflect a positive correlation between DIN and DRP. In contrast, *D. geminata* proliferations can be growth‐limited when DIN concentrations are very low (Kilroy & Larned, 2016), leading to a positive correlation between *D. geminata* abundance and DIN. In the present analysis, we focused on the potential effect of increases in DIN at these low levels.

Three South Island sites with mean DIN close to or below the 10 mg/m^3^ threshold below which cell division and stalk production may be limited (Kilroy & Larned, 2016) also had low DRP (CH1, TK4, and DN10; see Tables 2 and 4). Percentage cover by *D. geminata* was highest at TK4, followed by CH1; cover was never observed at DN10. There was no evidence for an increase in DIN between 1995 and 2006 at these sites and, on the basis of DRP only, we would expect high cover at all three. Between 2006 and 2015, mean annual DIN at DN10 was always lower than the suggested 10 mg/m^3^. Low DIN may therefore partially explain the absence of visible *D. geminata* at that site, despite the fact that the site is in the dam‐regulated Monowai River. Dam‐regulated rivers are especially favorable for proliferations (Kirkwood, Jackson, & McCauley, 2009).

In a broader context, our findings for *D. geminata* in New Zealand provide further evidence that counters the proposal of ubiquity for freshwater diatoms in general (Finlay et al., 2002). Finlay et al. (2002) maintained that: “The argument in favor of endemic diatom species is untenable, because it is not possible to disprove their existence elsewhere in the biosphere”. We contend that our combined environmental and genetic evidence against the presence of *D. geminata* in New Zealand prior to about 2004 is close to qualifying as proof of the species’ absence in this particular part of the biosphere. The natural range of *D. geminata* likely extended across broad areas of the Northern Hemisphere (Blanco & Ector, 2009), and the species could be considered endemic to that region. However, extensive bloom formation was evidently rare, although blooms have been reported for many decades in Norwegian rivers (Lindstrøm & Skulberg, 2008). The absence of *D. geminata* from New Zealand until the early 2000s likely reflected its relative rarity in the Northern Hemisphere, until blooms started to become widespread in the late 1980s (Blanco & Ector, 2009), increasing the likelihood of human‐mediated, long‐distance transport of *D. geminata* cells. Once in New Zealand, *D. geminata*'s eventual distribution was determined by environmental suitability so that, currently, the species appears to be restricted to low‐phosphorus rivers, which occur mostly in the South Island.

The case of *D. geminata* in New Zealand raises the question of how many other diatom taxa are still arriving in new locations, accompanying the recent rapid increase in global trade and travel by both sea and air (Tatem, 2009). A further example in New Zealand may be the centric diatom *Lindavia intermedia* (Manguin ex Kociolek & Reviers) Nakov, Guillory, Julius, Theriot & Alverson ex W.C.Daniels, Novis & Edlund, recently identified as the cause of large‐scale mucilage production in lakes since the mid‐2000s (Novis, Schallenberg, Saulnier‐Talbot, Kilroy, & Reid, in press).

In conclusion, we find no evidence either from the time series of monthly DRP and DIN data, or from new molecular analyses, to support the ubiquity hypothesis for *D. geminata* (Taylor & Bothwell, 2014) in the New Zealand context. None of the data suggest that *D. geminata* was present in New Zealand freshwaters prior to the early 2000s, indicating that its initial treatment in 2004 as an introduced invasive species (Kilroy & Unwin, 2011) was justified. However, our data do support the proposal by Taylor and Bothwell (2014) that the dynamics of *D. geminata* proliferations in rivers over time are likely to be linked to changes in DRP concentrations. We also agree with Taylor and Bothwell (2014) that distinguishing native and non‐native invasive species is important, and note that our findings do not address invasion history of the species in areas of the Northern Hemisphere.

## AUTHOR CONTRIBUTIONS

CK performed data analysis and interpretation in the water chemistry component of the study; PN designed, executed and interpreted the molecular component of the study; CK and PN wrote and revised the manuscript.

## CONFLICT OF INTEREST

None declared.
