# Supplementary material for: Is Didymosphenia geminata an introduced species in New Zealand? Evidence from trends in water chemistry, and chloroplast DNA
Source: Ecol Evol. 2017 Dec 12;8(2):904–19. doi: 10.1002/ece3.3572 (PMC5773294; doi:10.1002/ece3.3572)
Supplement: Supplementary file 1 [file ECE3-8-904-s001.pdf]

SUPPORTING INFORMATION

Kilroy, C., Novis, P. Is *Didymosphenia geminata* an introduced species in New Zealand? Evidence from trends in water chemistry, and chloroplast DNA. Ecology & Evolution

Table S1. Site names and locations (New Zealand map grid coordinates) of the 77 river sites in New Zealand's national river water quality monitoring network. Sites highlighted in bold type are reference sites (draining largely unimpacted catchments). Status: bloom = persistent visible *D. geminata* cover; occ = occasional visible but not persistent *D. geminata* cover; neg = negligible *D. geminata* cover; nd = *D. geminata* not observed. For definitions refer to Methods section in main text. Mean values for DRP and DIN are shown for the pre- and post-*D. geminata* (Dg) periods (1989-2005 and 2006-2015, respectively).

						DRP ($mg\ m^{-3}$)		DIN ($mg\ m^{-3}$)	
Code	Site name	E	N	Status	Date of first detection	pre-Dg	post-Dg	pre-Dg	post-Dg
**North Island sites**									
**WH1**	**Waipapa at Forest Ranger**	2573046	6658281	nd		4.7	4.6	23.3	18.4
WH2	Waitangi at Wakelins	2606139	6657724	nd		5.4	4.7	183.1	133.5
WH3	Mangakahia at Titoki Br	2605914	6607059	nd		6.5	5.9	86.1	51.4
WH4	Wairua at Purua	2614941	6615862	nd		19.4	17.5	399.2	201.2
AK1	Hoteo at Gubbs	2646033	6534038	nd		18.7	17.0	275.7	150.9
AK2	Rangitopuni at Walkers	2654991	6494819	nd		17.2	16.6	206.6	143.2
**HM1**	**Waipa at Otewa**	2715637	6323441	nd		9.4	8.7	241.8	227.2
HM2	Waipa at Whatawhata	2699656	6376025	nd		21.5	18.4	709.8	693.2
HM3	Waikato at Ham Traff Br	2711831	6376412	nd		11.2	14.4	165.7	249.1
HM4	Waikato at Rangiriri	2698887	6416748	nd		21.6	21.8	310.2	378.9
HM5	Waihou at Te Aroha	2749432	6402603	nd		54.9	48.9	1116	1127
**HM6**	**Ohinemuri at Karangahake**	2750577	6417205	nd		6.7	2.6	337.1	344.3
**RO1**	**Tarawera at Lake Outlet**	2817441	6330344	nd		2.2	3.1	2.2	2.0
RO2	Tarawera at Awakaponga	2841208	6355733	nd		70.1	65.1	405.9	443.9
RO3	Rangitaiki at Murupara	2832905	6298355	nd		21.5	20.6	598.4	893.1
**RO4**	**Whirinaki at Galatea**	2837043	6295952	nd		20.2	19.7	105.5	65.9
RO5	Rangitaiki at Te Teko	2843629	6344416	nd		15.5	11.6	329.4	385.8
**RO6**	**Waikato at Reids Farm**	2778271	6277678	nd		1.1	1.4	2.0	1.9
TU1	Whanganui at Te Maire	2699812	6248985	nd		6.7	6.1	195.5	160.0
**TU2**	**Tongariro at Turangi**	2753749	6241697	nd		12.5	13.9	32.2	31.3
WA1	Waitara at Bertrand Rd	2618682	6238934	nd		5.3	5.8	242.2	238.0
**WA2**	**Manganui at SH3**	2618865	6213047	nd		10.0	8.9	92.3	79.7
WA3	Waingongoro at SH45	2614004	6180263	nd		45.2	46.3	1892	1700
WA4	Wanganui at Paetawa	2693722	6156603	nd		4.7	4.1	117.1	101.0

Code	Site name	E	N	Status	Date of first detection	DRP (mg m^{-3})		DIN (mg m^{-3})	
						pre-Dg	post-Dg	pre-Dg	post-Dg
**WA5**	**Rangitikei at Mangaweka**	2750370	6151340	nd		4.1	3.6	66.7	41.9
WA6	Rangitikei at Kakariki	2718305	6117218	nd		4.5	4.2	59.5	34.3
**WA7**	**Manawatu at Weber Rd**	2775061	6102713	nd		9.2	9.6	398.8	305.0
WA8	Manawatu at Teachers Coll	2733100	6089200	nd		9.0	7.4	521.9	285.8
WA9	Manawatu at Opiki Bridge	2734300	6089200	nd		32.2	15.0	659.9	492.2
GS1	Waipaoa at Kanakanaia	2935881	6292331	nd		2.6	3.2	77.5	68.4
**GS2**	**Waikohu at No 1 Bridge**	2908293	6299695	nd		7.0	8.6	157.9	128.7
GS3	Motu at Waitangirua	2914723	6323286	nd		8.3	9.0	81.3	66.0
GS4	Motu at Houpoto	2918077	6360863	nd		10.9	10.6	39.5	32.8
**HV1**	**Makaroro at Burnt Br**	2792800	6148800	nd		4.8	4.8	46.3	43.1
HV2	Tukituki at Red Bridge	2846597	6158135	nd		7.5	7.0	442.2	353.5
HV3	Ngaruroro at Chesterhope	2842492	6171513	nd		6.4	6.8	52.9	61.7
**HV4**	**Ngaruroro at Kuripapango**	2796945	6197400	nd		1.8	2.1	8.7	9.4
HV5	Mohaka at Raupunga	2867237	6228523	nd		8.2	6.1	64.3	89.6
**HV6**	**Mohaka at Glenfalls**	2823979	6218769	nd		5.6	4.8	155.6	244.4
WN1	Hutt at Boulcott	2671222	5999194	nd		6.4	4.2	240.3	194.6
**WN2**	**Hutt at Kaitoke**	2694176	6015002	nd		3.6	3.7	35.2	29.4
WN3	Ruamahanga at Waihenga	2714559	5998412	nd		13.3	12.2	397.8	334.8
WN4	Ruamahanga at Wardells	2734728	6019152	nd		9.5	9.1	568.2	552.3
**WN5**	**Ruamahanga at SH2**	2729914	6046092	nd		2.8	2.7	38.4	32.3
**South Island sites**									
NN1	Motueka at Woodstock	2495112	5994300	occ	Feb-07	2.7	3.0	124.7	159.6
**NN2**	**Motueka at Gorge**	2502787	5952647	nd		2.4	2.8	22.6	24.2
**NN3**	**Wairau at Dip Flat**	2503477	5923767	occ	Mar-08	2.8	3.5	12.8	13.6
NN4	Wairau at Tuamarina	2590635	5973743	neg	Oct-09	3.4	3.9	62.6	90.6
**NN5**	**Buller at Longford**	2458970	5937980	bloom	Nov-05	1.1	1.4	25.8	29.6
GY1	Buller at Te Kuha	2401960	5929475	occ	Jan-09	1.9	1.9	47.2	70.0
GY2	Grey at Dobson	2369993	5860128	nd		2.3	2.6	89.5	142.4
**GY3**	**Grey at Waipuna**	2410022	5872000	occ	Jun-08	2.0	2.1	30.0	38.4
**GY4**	**Haast at Roaring Billy**	2212857	5689490	neg	Feb-07	1.2	1.3	32.6	32.6
**CH1**	**Hurunui at Mandamus**	2472514	5824007	bloom	Apr-07	1.1	1.3	12.0	12.1
CH2	Hurunui at SH1 Bridge	2517897	5812110	occ	Nov-07	2.8	2.6	281.7	363.3
**CH3**	**Waimakariri at Gorge**	2433142	5760479	neg	Jan-10	2.0	2.1	63.0	82.0
CH4	Waimakariri u/s Old Hw Br	2481804	5754678	nd		1.9	2.1	75.2	164.7
TK1	Opihi at Waipopo	2376168	5658589	occ	Dec-07	3.2	3.8	362.0	492.0
TK2	Opihi at Rockwood	2345438	5669013	occ	Jan-14	3.8	4.5	702.2	1103
**TK3**	**Opuha at Skipton Br**	2348185	5679030	bloom	Jan-08	2.2	1.6	242.9	237.6

						DRP (mg m^{-3})		DIN (mg m^{-3})	
**Code**	**Site name**	**E**	**N**	**Status**	**Date of first detection**	**pre-Dg**	**post-Dg**	**pre-Dg**	**post-Dg**
**TK4**	**Waitaki at Kurow**	2308015	5608817	bloom	Jan-06	0.6	0.7	12.7	7.9
TK5	Hakataramea u/s MH Br	2311170	5606221	occ	Nov-07	3.1	3.7	21.3	28.8
TK6	Waitaki at SH1 Bridge	2360000	5585000	bloom	Jan-06	2.0	1.1	86.9	47.6
**AX1**	**Clutha at Luggate Br**	2215500	5601900	bloom	Sep-05	0.5	0.6	39.4	30.1
**AX2**	**Kawarau at Chards Rd**	2184376	5569787	bloom	Jan-07	0.7	1.4	31.2	35.5
**AX3**	**Shotover at Bowens Peak**	2172199	5571035	bloom	Apr-07	0.9	0.9	21.1	17.0
AX4	Clutha at Millers Flat	2230200	5498800	bloom	Sep-06	0.7	0.9	40.4	33.2
DN1	Taieri at Tiroiti	2295855	5546607	nd		14.0	12.1	24.8	31.4
**DN2**	**Sutton at SH87**	2283172	5508439	nd		5.2	5.0	26.1	17.4
DN3	Taieri at Outram	2295809	5481042	nd		9.0	7.9	42.8	50.8
DN4	Clutha at Balclutha	2258996	5436246	bloom	Feb-07	1.7	2.0	68.8	62.3
DN5	Mataura at Seaward Downs	2186569	5416006	neg	May-07	15.8	11.3	1030	1187
**DN6**	**Mataura at Parawa**	2163536	5507277	nd		5.5	5.7	232.8	283.6
**DN7**	**Oreti at Lumsden**	2154070	5489203	neg	Oct-05	2.6	2.8	419.3	608.4
DN8	Oreti at Riverton Hwy Br	2145365	5420798	nd		6.0	5.9	848.5	1078
DN9	Waiau at Tuatapere	2099363	5439848	bloom	Oct-04	2.1	1.8	179.2	235.1
**DN10**	**Monowai below Gates**	2085337	5475050	nd		0.5	0.6	9.3	6.9

TABLE S2. Voucher information for strains used in the molecular study, and Genbank accession numbers for the resulting sequences.

Site	Collection date	Voucher	Country/region	Genbank accession numbers		
				**atpF-atpH**	**rbcS-rbcL**	**secA-rbcR**
Gowan River	11 December 2012	CHR589900	New Zealand/Nelson	KY777671	KY777682	KY777691
Buller River at Howard Junction	11 December 2012	CHR589901	New Zealand/Nelson	KY777674	KY777685	KY777694
Buller River at top bridge	11 December 2012	CHR589902	New Zealand/Nelson	KY777675	KY777686	KY777695
Hurunui at SH7	10 December 2012	CHR589903	New Zealand/Canterbury	KY777672	KY777683	KY777692
Wye River	February 2011	CHR589904	New Zealand/Southland	KY777676	KY777687	KY777696
Mararoa River at Kiwi Burn	7 November 2011	CHR589905	New Zealand/Southland	KY777673	KY777684	KY777693
Little Qualicum River	7 April 2006	CHR589906	Canada/British Columbia	KY777677	KY777688	KY777697
Boulder Creek	Unknown	NA	USA/Colorado	KC509523	KC509523	KC509523
Boulder Creek	15 May 2006	CHR640983	USA/Montana	KY777679	KY777689	KY777699
River Coquet	4 May 2006	CHR641004	UK	KY777678	KY777690	KY777698
Nidelva River	20 September 2006	CHR640997	Norway	KY777681	NS	KY777701
Tar River	October 2008	CHR641005	Iran	KY777680	See text	KY777700
